# Adolescent sadfishing on social media: anxiety, depression, attention seeking, and lack of perceived social support as potential contributors

**DOI:** 10.1186/s40359-023-01420-y

**Published:** 2023-11-07

**Authors:** Reza Shabahang, Hyejin Shim, Mara S. Aruguete, Ágnes Zsila

**Affiliations:** 1 University of Tehran, Tehran, Iran; 2 Laureate Institute for Brain Research, Oklahoma, OK USA; 3 Lincoln University, Missouri, MO USA; 4 Institute of Psychology, Pázmány Péter Catholic University, Budapest, Hungary; 5 Institute of Psychology, ELTE Eötvös Loránd University, Budapest, Hungary

**Keywords:** Adolescents, Anxiety, Depression, Self-disclosure, Social media, Social support

## Abstract

**Background:**

Sympathy-seeking negative online self-disclosure, or “sadfishing,” has proliferated in social media. This study investigates sadfishing by developing and validating a brief self-report questionnaire of the construct and exploring potential psychological correlates.

**Methods:**

A total of 345 Iranian adolescent social media users (*M*_*age*_ = 16.29, *SD*_*age*_ = 1.52) participated in the study. Participants completed the newly constructed *Social Media Sadfishing Questionnaire*, in addition to measures of anxiety, depression, attention seeking, perceived social support, and social media use integration.

**Results:**

Factor analyses revealed a unidimensional structure of the 5-item *Social Media Sadfishing Questionnaire*. The questionnaire yielded sound construct validity and internal consistency. Anxiety, depression, and attention seeking were positively associated with sadfishing, while perceived social support from family and friends was negatively associated with sadfishing. Negative online reactions to sadfishing were rare. Boys reported higher sadfishing tendencies than girls at age 12; however, sadfishing in boys declined at a higher rate than in girls with age.

**Conclusions:**

The findings suggest that negative affect and attention seeking, combined with feelings of low social support, can be associated with adolescent sadfishing on social media. The quantitative results shed new light on the contribution of psychosocial factors to sadfishing.

## Background

Today’s adolescents were born and raised in a technology-reliant world. Consistent use of social media is integral part of adolescent culture. Using social media is the most popular leisure activity for adolescents and, though they tend to evaluate their social media use as nonproblematic, many adolescent social media users are at risk of social media addiction [1]. In a recent large-scale study by Chegeni et al. [2], Iranian participants used social media for 4 h daily on average, mostly WhatsApp, Telegram, and Instagram. The risk of social media addiction was the highest among adolescents (26.3%), which makes the evaluation of social media behaviors of Iranian users worthwhile [2]. This study examines sympathy-seeking negative online self-disclosure, or “sadfishing” in an Iranian adolescent sample. Though such self-disclosure is common on social media, quantitative investigations of the phenomenon have been rare. One purpose of the present study is to introduce a scale to measure sadfishing on social media and test its psychometric properties. A second purpose is to identify psychosocial predictors (i.e., anxiety, depression, attention seeking, social media use integration, and perceived social support) of sadfishing.

Social media enables users to self-disclose personal information to a wide variety of others, sometimes with excessive breadth and depth (social media oversharing; 3). Self-disclosure is pervasive among adolescents and considered to be a crucial interpersonal process, enhancing the attainment of key developmental milestones [3]. Similar to offline self-disclosure, online self-disclosure fulfills adolescent social needs, and for some (e.g., highly anxious adolescents), online self-disclosure is preferable to offline self-disclosure [4]. Luo and Hancock [5] argued that social media self-disclosure and well-being are reciprocally related. Social media self-disclosure positively affects well-being through perceived connectedness, social support, capitalization (increasing the salience of an event and restructuring memory), and perceived authenticity. In turn, enhanced well-being affects social media self-disclosure by increasing interpersonal (e.g., relational maintenance) and intrapersonal (self-expression and identity clarification) motivations. Though previous studies have found that social media self-disclosure contributes to life satisfaction and wellbeing [6, 7], it can also elevate the risk of negative outcomes including cyberbullying victimization [8], stalking and cyberstalking, sexual abuse, criminal exploitation (e.g., identity theft), government surveillance, and interpersonal harms (e.g., critical feedback, stigma; 10).

Social media allows users to post both positive and negative emotional content. Previous studies suggest that there is a positivity bias in social media posts [9] and users consider expression of positive emotions (i.e., joy and pride) more appropriate than negative emotions (i.e., anger, sadness, and worry; 12). Individuals are more likely to share positive than negative events [10] and prefer to hide negative content in public nondirected communication (posts that a group of people can see [e.g., followers]; 14, 15), compared to directed communication (e.g., interacting via private message with a specific person).Vermeulen et al. [11] found that adolescents show distinctive patterns of emotion sharing on different social media platforms (e.g., Facebook, Instagram, and Snapchat for positive emotions, and Twitter for negative emotions). Vermeulen et al. [12] proposed that the decision to share emotions online or offline (i.e., face-to-face, texting, and calling) is influenced by the valence, type, and intensity of the emotion, in addition to the affordances of the mode, social norms, and impression management concerns.

Despite social risks and impression concerns of online sharing of negative emotions [7, 13], some users opt to share personal struggles on social media (e.g., physical and mental health concerns, relationship problems, traumatic experiences; 19). Studies examining negative online self-disclosure suggest that some people self-disclose the acts they regret to obtain guilt relief [14] while others are motivated to engage in mutual storytelling about difficult experiences [15]. Users may disclose their stigmatized experiences on social media (e.g., women who have experienced abortion or miscarriage) which could result in positive individual (e.g., feelings of relief), dyadic (social bonding), and network (e.g., facilitation of others’ disclosures) outcomes [16]. Information about stigmatizing experiences can evoke specific reactions in other users, such as positive social support, reciprocal disclosure, and criticism [17].

The present study investigates one type of negative social media self-disclosure among adolescents: sadfishing. The term “sadfishing” was recently introduced to describe posting about personal struggles to seek attention and sympathy online [18]. Sadfishing can be defined as intentionally posting details about emotional difficulties, feelings of being misunderstood, and interpersonal challenges on social media with the purpose of evoking sympathy and attention from the online community. Previous investigations [18–20] have identified the main characteristics of sadfishing posts such as containing negative content, being personal, and intending to evoke sympathy. The intention to gain sympathetic feedback distinguishes sadfishing from the broader construct of negative self-disclosure. Sadfishing appears to be increasing among youth [21].The present study examines whether sadfishing is associated with perceived social support, negative affect, attention seeking, and excessive use of social media.

One motivation for sadfishing may be to increase perceived social support. Emotional posts are often shared online during or shortly after an emotional experience [22, 23] with the intent of evoking supportive feedback from others [24]. Preceding the popularity of social media, individuals sought emotional support by sharing their personal problems with those closely associated with them (e.g., family members, friends, 31), while self-disclosure to strangers was considered an inappropriate behavior [25]. Increased use of social media has opened a new avenue for youth to express negative feelings [26, 27]. The *Social Compensation Hypothesis* [28] postulates that users who have few face-to-face intimacies benefit from using computer-mediated communication. The hypothesis also suggests that social media can compensate for face-to-face relationships as it provides social support during life’s challenges. Drawing on this hypothesis, users with lack of perceived social support would be expected to exhibit sadfishing more than those with greater social support.

Negative affect may also influence sadfishing. Previous studies have suggested that adolescents with mental health concerns (e.g., depression, anxiety) have higher tendency to use social media frequently [29, 30], and are more likely to communicate with strangers online [31]. For adolescents with psychological distress, receiving positive feedback and emotional support in social media can be rewarding [32–34], and may contribute to the maintenance of mental health in some cases despite negative life events [32]. Indeed, individuals can derive gratification by simply talking about themselves (see 39 for a review); thus, some people dedicate considerable time to sharing personal information on social media to gain immediate social reward [32, 33]. Petrofes et al. [19] classified sadfishers and non-sadfishers through open-ended responses to the question “under what circumstances would you feel compelled to exaggerate your personal mental health status online?” and compared the two groups based on mental health indicators. They found greater endorsement of anxious attachment among sadfishers compared to non-sadfishers, though the groups did not differ in perceived social support [19]. Petrofes et al. [19] concluded that sadfishing is associated with persistent trait anxiety rather than perceived lack of social support. Given the evidence, we expect that adolescents reporting depression and anxiety will show a higher tendency for sadfishing.

Attention seeking may additionally contribute to sadfishing. Emotional expression on social media appears to attract more attention than other types of posts [6, 35]. In fact, negative self-disclosure may be posted with the purpose of going viral on social media [36]. In a qualitative study, Ramadhani et al. [20] investigated sadfishing on TikTok. Results indicated that sadfishing was motivated by gaining sympathy as well as creating content which is popular among other users. The study concluded that sadfishing is related to attention seeking (i.e., desire for posts going viral; 26). Attention seeking is also related to adolescent social media users’ excessive breadth and depth of sharing (oversharing) on social media [37]. Finally, Hawk et al. [38] demonstrated that adolescent narcissism was associated with social media disclosure and problematic social media use via increased attention seeking. Therefore, we expect that attention seeking will be positively associated with sadfishing.

Finally, extensive social media use may be associated with sadfishing. Among excessive users, self-disclosure may be an impulsive behavior [39]. Psychological and emotional dependency on social media has the potential to increase social media self-disclosure [40]. Research shows that problematic social media use is associated with adolescent users’ oversharing [37]. Hence, extensive use of social media (i.e., social media integration and emotional connection) may be associated with sadfishing.

The present study examines sadfishing and its psychosocial correlates in adolescents. Considering adolescents’ age-related life challenges and vulnerability to distress [41], high tendency for self-disclosure [3], strong peer influence [42], and high emotional engagement with social media [43], it is not surprising that sadfishing is common among adolescents [21]. About 13% of teens post about their personal problems on social media [44]. Such sharing behavior could lead to online bashing victimization, stigmatization, cyberbullying victimization, cyberstalking, sexual abuse, and criminal exploitation (e.g., identity theft). To date, research on sadfishing has been limited. Quantitative analysis of sadfishing may be hindered by the lack of a psychometrically sound questionnaire for measuring the construct. The present study seeks to fill this gap in the literature by introducing a brief, sound questionnaire measuring sadfishing, the *Social Media Sadfishing Questionnaire*, and testing the psychometric properties of the measure. Furthermore, we extend the scope of previous investigations by examining affective background variables and social media use patterns in one comprehensive model to gain a more nuanced picture of the role of affective and behavioral factors that can explain sadfishing based on previous knowledge. A recent study on the predictors of sadfishing used a sample of US college students [19]. The present study also extends the scope of previous studies on sadfishing to a culturally different context, which may provide preliminary findings on possible similarities and differences across cultures in the phenomenon of sadfishing. Based on the literature, we propose the following hypotheses: (i) perceived social support will be negatively associated with sadfishing, and (ii) negative affect (anxiety and depression), attention seeking, and extensive use of social media (i.e., social media integration and emotional connection) will be positively associated with sadfishing.

## Methods

### Participants

The convenience sample consisted of Iranian adolescent social media users recruited from local high schools in Rasht, Guilan, Iran. Data collection was conducted during May and June 2021. A total of 345 adolescent social media users (263 girls and 82 boys; *M*_*age*_ = 16.29, *SD*_*age*_ = 1.52) participated in the study. To participate, respondents were required to be active on at least one popular social media platform (e.g., Instagram, Facebook, and/or Twitter), and post on social media at least once a week. All procedures were performed in accordance with the relevant guidelines and regulations of the Helsinki Declaration. This study was conducted with the ethical approval of the Institutional Review Board of Guilan University. Online informed consent was obtained by the parents or legal guardians of participants, as well as the participants themselves. Participants and parents/guardians were informed about the purpose of the study, the anonymity of the data collection procedure, confidentiality and privacy of the data, and the full right to discontinue or refuse to participate at any time. Answering all scales of the survey was necessary for inclusion in the data analysis. The survey took approximately 10 min to complete. Participants received book discount cards in compensation for participation.

### Measures

The *Social Media Sadfishing Questionnaire* was developed for the present study to assess adolescents’ tendency to post details about emotional difficulties, feelings of being misunderstood, and interpersonal challenges to evoke sympathy and attention of their online community (8 items; e.g.,“I post my emotional pain on social media to get support from others”; see Table 1). The items were generated based on literature of self-disclosure (e.g., 51), offline and online adolescent self-disclosure [4], social media self-disclosure mechanisms [5], negative self-disclosure [14], sadfishing [18–20], and previous measures of social media self-disclosure (e.g., 3). In-depth unstructured online interviews, expert suggestions, and pilot-testing were also conducted in the process of item generation and revision. The number of items of the *Social Media Sadfishing Questionnaire* was low to [1] specifically measure sympathy-seeking negative online self-disclosure and [2] reduce the confusion and cognitive overload in adolescent respondents. Lengthy questionnaires have been associated with poor response rate, high attrition, and low quality of data obtained. Studies have supported the functionality and viability of brief and even single-item measures of psychological constructs, especially when constructs are well-defined and narrow in scope [45]. Items were rated from 1 (*strongly disagree*) to 5 (*strongly agree*), with higher scores indicating higher social media sadfishing tendencies. The construct validity, factor structure, item characteristics, and internal consistency of the *Social Media Sadfishing Questionnaire* were investigated in this study.

The *Brief Symptom Inventory* (*BSI*; 53) contains 53 items that assess nine symptom dimensions including somatization, obsession-compulsion, interpersonal sensitivity, depression, anxiety, hostility, phobic anxiety, paranoid ideation, and psychoticism. Response options range from 0 (*not at all*) to 4 (*extremely*), where higher scores indicate higher intensity of distress during the past seven days. Since previous studies provided evidence on the association of sadfishing [19] and excessive social media use [29, 30] with symptoms of depression and anxiety, and the present study’s aim was to assess negative affect in subclinical qualities instead of more specific psychiatric disorders (e.g., paranoid ideation or psychoticism), which may not be prevalent in general population samples, the depression (6 items; e.g., “During the past 7 days, how much were you distressed by feeling lonely?”) and anxiety subscales (6 items; e.g., “During the past 7 days, how much were you distressed by feeling tense or keyed up?”) were used. The measure yielded acceptable Cronbach’s alpha values (0.71 to 0.85) and test-retest reliability in a 2-week interval (0.68 to 0.91). Significant correlations of the *BSI* dimensions with *MMPI* subscales (r*s* over 0.30) confirmed convergent validity [46]. The Persian version of the *BSI* was shown to have satisfactory construct validity (CFA fit indices were CFI = 0.97, NFI = 0.97, RMSEA = 0.077) and reliability (*α* = 0.71 to 0.87; 55).

The *Brief Histrionic Personality Scale* (*BHPS*; 56) is an 11-item self-report questionnaire designed to assess histrionic personality by two subscales including Seductiveness (6 items) and Attention Seeking (5 items). The Seductiveness subscale (e.g., “I find it exciting to flirt with others”) was unrelated to our research questions and is inappropriate for our sample (based on cultural norms and age restrictions). Therefore, similar to Shabahang et al. [37], we administered only the Attention Seeking subscale in the present study (e.g., “I like to be the center of attention”). Responses are scored on a 4-point Likert scale (1 = *never true*, 4 = *always true*). The Attention Seeking subscale was found to be significantly associated with measures of extraversion (r = .42) and openness (r = .27), confirming convergent validity [47]. The Attention Seeking subscale demonstrated high reliability in an Iranian sample (*α* = 0.89; 57).

The *Multidimensional Scale of Perceived Social Support* (*MSPSS*; 58) is a 12-item scale that measures perceived social support from three sources, namely family (4 items; items of 3, 4, 8, & 11; e.g., “My family really tries to help me”), friends (4 items; items of 6, 7, 9, & 12; e.g., “I can talk about my problems with my friends”), and significant others (4 items; items of 1, 2, 5, & 10; e.g., “There is a special person who is around when I am in need”), with responses scored on a 7-point Likert scale (1 = *very strongly disagree*, 7 = *very strongly agree*). Zimet et al. [48] reported satisfactory psychometric properties of the measure. High Cronbach’s alpha values were obtained for the total scale (α = 0.88) and three subscales comprising Family (*α* = 0.87), Friends (*α* = 0.85), and Significant Others (*α* = 0.91; 58). The negative correlation of the *MSPSS* with measures of anxiety (*r* = − .24 for the Family subscale only) and depression (*r*s = − 0.13 to − 0.24) confirmed the validity of the *MSPSS* [48]. The Persian version of the *MSPSS* showed high reliability (*α* = 0.85 to 0.93) and test-retest reliability in the general population (*r*s = 0.74 to 0.84) [49].

The *Social Media Use Integration Scale* (*SMUIS*; 60) is a 10-item scale measuring social media users’ social integration and emotional connection (*SIEC*; 6 items) and integration into social routines (*ISR*; 4 items), with response options ranging from 1 (*strongly disagree*) to 6 (*strongly agree*). An example is “I enjoy checking my social media accounts.” Jenkins-Guarnieri et al. [50] found evidence for a two-dimensional structure of the *SMUIS* with strong internal consistency of the total scale (*α* = 0.91) and the subscales (*SIEC*’s α = 0.89, *ISR*’s α = 0.82). The high positive correlation with a previously published social media use measure (Facebook use intensity) provided evidence for convergent validity of the *SMUIS* (r = .70 to 0.75; 60). The validity and reliability of the Persian version of the *SMUIS* has also been established [51].

The items “How often do you receive critical feedback following posting about personal struggles and negative emotional states on social media?” and “How often do you feel unsupported following posting about personal struggles and negative emotional states on social media?” were included to investigate negative feedback following online sadfishing. Response options were provided on a 5-point Likert type scale from *not at all* [1] to *always* [4].

### Procedure

We initially conceptualized sadfishing by reviewing previous theoretical and empirical studies discussing self-disclosure [52], offline and online adolescents’ self-disclosure [4], social media self-disclosure mechanisms [5], negative self-disclosure [14], and sadfishing [18–20]. Following the initial generation of the items, a series of unstructured in-depth, online interviews were conducted with six adolescent social media users (*M*_*age*_ = 15.67, *SD*_*age*_ = 1.50) who self-reported posting about personal challenges on social media with the intention of receiving attention, support, and sympathy. Interviewees were recruited through a post on one of the social media channels of a high school in Rasht, inviting them to participate in a study concerning social media negative self-disclosure. The interviews were conducted in order to improve the content of the developed items generated using the sadfishing literature and self-disclosure measures. The unstructured, open interview was centered around definitions of individual challenges and emotional difficulties, online disclosure, expression of personal life crises in the social media sphere, sympathy and attention seeking via dramatic posts, user’s sentiments following posting, and followers’/viewers’ reactions to posts. Common topics, ideas, and patterns in responses were identified to draw a preliminary conclusion about themes of sadfishing and compatibility of these themes with the developed items. Adolescents’ responses in the interview phase helped further refine the construct of social media sadfishing and the items of the *Social Media Sadfishing Questionnaire*. In an online process, a panel of six experts in media psychology and communication science having experience in scale development using adolescent samples assessed the generated items based on clarity, simplicity, and relevance. The experts were academics with doctoral degrees in psychology, working in academic centers in Guilan, Iran. After making minor modifications based on experts’ recommendations, the item-content validity index (I-CVI) was assessed for each item by dividing the number of experts judging the item as very relevant by the total number of experts (> 0.79 = the item is relevant; 0.70 − 0.79 = needs revision; and < 0.70 = needs to be eliminated). The scale-level content validity index (S-CVI/Ave) was computed by taking the sum of the I-CVIs divided by the total number of items,with ≥ 0.90, indicating excellent content validity of the questionnaire [53]. Next, an online pilot study was conducted on a sample drawn from the population of interest (adolescent social media users, high school students of Rasht city; *n* = 30, 15 girls) to check the scale’s language, participants’ understanding of items, item difficulty, and completion time. Respondents were asked to rate the level of difficulty in understanding each of the items (0 = *Completely incomprehensible*, 3 = *Totally understandable*) and to respond to an open-ended question of “*How could the questionnaire items you answered be improved? Please write your understanding of each item. If you have any difficulty in understanding the wording of any items, point it out and suggest a replacement*” after completing the *Social Media Sadfishing Questionnaire*. Minor modifications were made to item wording in accordance with the pilot investigation (participants’ ratings of perceived difficulty, misunderstandings, and suggestions).

Finally, the link to the online survey, including the final *Social Media Sadfishing Questionnaire* and additional measures of anxiety, depression, attention seeking, perceived social support, social media use integration, receipt of critical feedback, and unsupported feelings, was posted on the main social media channels of the eight high schools in Rasht, Guilan, Iran. After randomly selecting eight high schools in the city and making the necessary arrangements with the school authorities, the survey link was posted on the schools’ main social media channels (e.g., Instagram) by the school staff. These channels were maintained by the school staff and were designed to inform and communicate with students. Student membership and following these channels was mandatory, though the participation in the current study was voluntary. School identification numbers were collected with the sole purpose of ensuring that data were gathered from the intended population. All respondents and their parents or legal guardians provided online written informed consent to participate in the study.

### Statistical analysis

Exploratory Factor Analysis (EFA) and Confirmatory Factor Analysis (CFA) were conducted to evaluate construct validity of the *Social Media Sadfishing Questionnaire*. The following cutoff points were considered for fit indices of the CFA: ≥ 0.90 for the comparative fit index (CFI; 63), ≥ 0.90 for the Tucker Lewis index (TLI; 64), and ≤ 0.08 for the root mean square error of approximation (RMSEA; 65).

For more accurate results (split-data strategy; 66), the responses (*N* = 345) were split in half randomly for EFA (*n* = 172) and CFA (*n* = 173). There is no general rule concerning sufficient sample size for executing structural modeling (SEM) techniques [54]. Even a sample consisting of fewer than 50 responses can yield interpretable results in factor analyses [55]. In a review of SEM studies, Kline [56] found 200 responses to be the median sample size. Comrey and Lee [57] provided a guideline for sample size adequacy for factor analysis: 50 as very poor, 100 as poor, 200 as fair, and more than 300 as good. The 10 cases per indicator variable method is also widely applied [58, 59]. According to Comrey and Lee’s [57] guideline for sample size adequacy, the sample size of the two sub-samples for EFA and CFA in the current study are between poor and fair. However, considering recommendations by Nunnally & Bernstein [58] and de Winter et al. [55], the two subsamples of 173 and 172 exceed the required sample size for conducting EFA and CFA on the 8-item *Social Media Sadfishing Questionnaire*.

The total sample (*N* = 345) was used to (1) evaluate the internal consistency of the *Social Media Sadfishing Questionnaire* and (2) investigate the predictors of sadfishing. The internal consistency/reliability of *Social Media Sadfishing Questionnaire* was examined calculating inter-item correlation, corrected item-total correlation, and Cronbach’s alpha. To investigate the associations between sadfishing and demographic/psychosocial variables, Pearson correlation and multiple regression analysis were conducted.

Data analysis was performed using SPSS statistical software [60] and the lavaan package [61] in R software [62]. Post-hoc power analysis was performed using G*Power software [63]. According to this analysis, effect sizes (r) above 0.15 have sufficient power (1-β) = 0.80 for correlations (α = 0.05, *N* = 345). The multiple regression analysis with the maximum number of predictors in our study (*n* = 8) showed strong statistical power (1.00) with the present sample size (*N* = 345) and effect size (f^2^ = 0.14) derived from the R^2^.

## Results

A S-CVI/Ave of 0.91 was obtained for the *Social Media Sadfishing Questionnaire* confirming excellent content validity of the questionnaire. Based on Cronbach’s alpha (*α* = 0.89, 95% CI = [0.87, 0.91]) and the results of initial factor and item analyses, three redundant items (items 1, 2, and 7) were deleted. Specifically, items 1 and 2 did not load to the same factor, while item 7 exhibited unexpectedly high residuals (> 0.05) with items 4, 5, and 8. The internal consistency of the *Social Media Sadfishing Questionnaire* with remaining items was excellent (5 items; α = 0.85, 95% CI = [0.82, 0.87]). The random half data (*n* = 172) were used for EFA. The Kaiser-Meyer-Olkin (*KMO* = 0.82) and Bartlett’s test of sphericity (*χ*^*2*^[10] = 356.664, *p* < .001) indicated the suitability of data for factor analyses. EFA results illustrated the one-factor structure of the *Social Media Sadfishing Questionnaire*, accounting for 63.01 of the total variances (see Table 1). The scree plot and eigenvalues supported that a large portion of variability loaded significantly on the general factor.

**Table 1:** Exploratory factor analysis of items in the *Social Media Sadfishing Questionnaire* (*n* = 172)

Item	MR1	MR2	*h* ^*2*^	*M*	*SD*
1. I share my painful experiences on social media to generate sympathy.	-0.01	0.9	0.8	0.2	-0.01
2. Social media provides an outlet for me to express my life challenges in a supportive environment to gain support.	0.07	0.56	0.37	0.63	0.07
**3. I post my emotional pain on social media to get support from others.**	0.72	0.07	0.59	0.41	0.72
**4. Social media is an outlet to share my bad experiences for gaining sympathy.**	0.41	0.25	0.36	0.64	0.41
**5. I seek compassion by posting my negative emotions on social media.**	0.81	-0.01	0.66	0.34	0.81
**6. I share the concerns of my life on social media to gain positive attention.**	0.86	-0.06	0.67	0.33	0.86
7. I display my complexities on social media to attract sympathetic reactions.	0.86	-0.01	0.73	0.27	0.86
**8. Posting my painful experiences on social media helps me to relieve tension through attracting sympathetic reactions.**	0.63	0.06	0.45	0.55	0.63

***Note.****h*^*2*^: communality (the amount of variance in the item/variable explained by the factor); *M*: mean; *SD*: standard deviation

Boldfaced items constitute the final, 5-item version of the *Social Media Sadfishing Questionnaire*

The other half of the dataset (*n* = 173) was used for CFA. CFA results confirmed the one-factor solution for the *Social Media Sadfishing Questionnaire* (*χ*^*2*^ = 10.424, *χ*^*2*^*/df* = 2.085; *CFI* = 0.986; *TLI* = 0.972; *RMSEA* = 0.079). CFA results satisfied the recommended benchmarks for good fit [64–66].

Finally, the total dataset (*N* = 345) was used to evaluate the reliability of the questionnaire. Item analyses showed excellent internal consistency in terms of inter-item correlation (range = 0.44 to 0.67), corrected item-total correlation (range = 0.59 to 0.73), and Cronbach’s alpha (*α* = 0.85, 95% *CI* = [0.82, 0.87]; see Table 2).

**Table 2:** Item characteristics, corrected item-total correlations, inter-item correlation, and Cronbach’s alpha of *Social Media Sadfishing Questionnaire* (*N* = 345)

Items	Mean (SD)		Scale mean if item deleted	Scale variance if item deleted	Corrected Item-Total Correlations	3	4	5	6	8
3	1.37	0.76	5.60	6.42	0.73	1				
4	1.47	0.83	5.50	6.61	0.59	0.52***	1			
5	1.26	0.68	5.71	6.95	0.67	0.61***	0.50***	1		
6	1.32	0.73	5.64	6.63	0.71	0.67***	0.44***	0.63***	1	
8	1.56	0.94	5.41	5.99	0.63	0.55***	0.49***	0.47***	0.56***	1

Cronbach’s alpha = 0.85.

***Note***. *: *p* < .05, **: *p* < .01, ***: *p* < .001.

After establishing the psychometric properties of the *Social Media Sadfishing Questionnaire*, Pearson correlation coefficients and multiple regression analysis were employed to thoroughly address the demographic and psychosocial correlates of sadfishing in adolescent social media users (*N* = 345).

A multiple regression analysis was conducted with gender and age to estimate their associations with adolescents’ social media sadfishing (see Table 3). It should be noted that, since the participants’ age range was 12–18 years, the intercept indicated the average social media sadfishing score of participants at age 12. First, for boys at age 12, the average social media sadfishing score was 9.93. For girls at age 12, the average social media sadfishing score was lower than boys by 3.05 (*p* = .006). Therefore, their social media sadfishing score was 6.88. In addition, for boys, the average social media sadfishing score decreased by 0.57 (*p* = .007) as age increased by 1 year. Interestingly, among girls, the average social media sadfishing score declined at a slower rate, specifically, 0.55 *less than* that of boys (*p* = .028).

**Table 3:** The interaction of age and gender in predicting online sadfishing (*N* = 345)

	*B*	*SE*	*β*	*p*-value
Intercept	9.926***	0.931		< 0.001
Girls	-3.048**	0.210	-0.415	0.007
Age	-0.571**	1.105	-0.275	0.006
Girls*Age	0.546*	0.247	0.392	0.028

***Note***.*B*: Unstandardized coefficient; *β*: Standardized coefficient

*: *p* < .05, **: *p* < .01, ***: *p* < .001

As hypothesized, results of correlational analysis (see Table 4) indicated that anxiety (*r* = .53, *p* < .01), depression (*r* = .55, *p* < .01), attention-seeking (*r* = .28, *p* < .01), *SMUIS* integration and emotional connection (*r* = .34, *p* < .01), and *SMUIS* integration into social routines (*r* = .18, *p* < .01) were positively correlated with social media sadfishing. Perceived social support from family (*r* = − .49, *p* < .01), friends (*r* = − .42, *p* < .01), and significant others (*r* = − .44, *p* < .01) were negatively correlated to social media sadfishing.

**Table 4:** Descriptive statistics and correlations for study variables (*N* = 345)

Variable	*M*	*SD*	1	2	3	4	5	6	7	8	9
1. Anxiety	7.04	7.09	**1**								
2. Depression	9.44	7.99	0.70**	**1**							
3. Attention seeking	11.40	3.20	0.19**	0.19**	**1**						
4. Perceived social support from family	19.54	7.82	− 0.49**	− 0.60**	− 0.01	**1**					
5. Perceived social support from friends	17.74	8.06	− 0.41**	− 0.45**	0.02	0.55**	**1**				
6. Perceived social support from significant others	19.30	8.29	− 0.42**	− 0.51**	0.03	0.75**	0.61**	**1**			
7. *SMUIS* Social integration and emotional connection	17.26	7.75	0.33**	0.39**	0.18**	− 0.30**	− 0.17**	− 0.25**	**1**		
8. *SMUIS* Integration into social routines	15.78	4.91	0.14*	0.18**	0.23**	− 0.10	0.03	− 0.05	0.64**	**1**	
9. Social media sadfishing	6.96	3.13	0.53**	0.55**	0.28**	− 0.49**	− 0.42**	− 0.44**	0.34**	0.18**	**1**
*Cronbach’s alpha*			0.93	0.94	0.72	0.91	0.92	0.92	0.86	0.80	0.85

***Note***.*SMUIS*: *Social Media Use Integration Scale*

*: *p* < .05, **: *p* < .01, ***: *p* < .001

Regression analysis showed that anxiety (*B* = 0.09,* β* = 0.21, *p* < .001), depression (*B* = 0.06,* β* = 0.14,* p* < .05), and attention seeking (*B* = 0.20, *β* = 0.20, *p* < .001) positively predicted social media sadfishing. In contrast, perceived social support from family (*B* = − 0.06, *β* = − 0.14, *p* < .05) and perceived social support from friends (*B* = − 0.06, *β* = − 0.15,* p* < .01) negatively predicted social media sadfishing. Despite the significant Pearson correlations, perceived social support from significant others, *SMUIS* integration and emotional connection, and *SMUIS* integration into social routines did not emerge as significant predictors of social media sadfishing in the regression analysis.The strongest predictor was attention seeking. These variables explained a considerable proportion of the total variance of social media sadfishing (∆*R*^*2*^ = 0.43; see Table 5).

**Table 5:** Summary statistics for the regression equation predicting social media sadfishing (*N* = 345)

Predictive Variables	*R* ^*2*^	*∆R* ^*2*^	*B*	*SD*	*β*	*t*	*p*
Final model	0.66	0.43					
**Anxiety**			0.09	0.03	0.21	3.55	**0.000**
**Depression**			0.06	0.02	0.14	2.18	**0.030**
**Attention seeking**			0.20	0.04	0.20	4.66	**0.000**
**Perceived social support from family**			− 0.06	0.03	− 0.14	-2.08	**0.039**
**Perceived social support from friends**			− 0.06	0.02	− 0.15	-2.73	**0.007**
Perceived social support from significant other			− 0.03	0.02	− 0.07	-1.04	0.298
Social media integration and emotional connection			0.03	0.02	0.08	1.44	0.150
Social media integration into social routines			0.01	0.03	0.01	0.33	0.739

***Note***. *R2*: R-squared or coefficient of determination; *∆R2*: Delta *R*^*2*^; *B*: Unstandardized coefficient; *β*: Standardized coefficient

Responses of participants to two questions of receiving critical feedback and feeling unsupported by the online community following sadfishing demonstrated a low percentage of negative reactions in response to sadfishing posts. Most adolescents reported that they never receive critical feedback (never = 75.1%; rarely = 13.3%; sometimes = 7.8%; very often = 2.0%; always = 1.7%) or feel unsupported by the online community (never = 64.9%; rarely = 12.8%; sometimes = 13.6%; very often = 5.8%; always = 2.9%) following sadfishing posts.

## Discussion

Posting personal challenges on social media to seek emotional support and positive attention has become popular among youth [26]. This online behavior is known as sadfishing. Studies of sadfishing have mainly employed qualitative research methods [18, 20]. Quantitative assessments and examination of correlates of sadfishing are scarce, especially in adolescent samples. The present study attempted to fill this gap by introducing a brief scale to assess adolescent sadfishing. After assessing the psychometric properties of the scale, we examined the demographic and psychosocial predictors of sadfishing.

First, we examined the psychometric properties of the *Social Media Sadfishing Questionnaire*. The 5-item unidimensional scale yielded good psychometric properties in terms of factor structure and internal consistency, suggesting that the scale is an appropriate tool to assess sympathy-seeking negative self-disclosure on social media. Future studies could possibly benefit from using this brief, valid questionnaire to investigate sympathy-seeking negative self-disclosure on social media among adolescents.

Two hypotheses were formulated regarding the psychosocial correlates of social media sadfishing among adolescents. As hypothesized, the regression analysis indicated that individuals with more symptoms of depression and anxiety, higher attention seeking, and lower perceived social support (from family and friends, but not from significant others) were more likely to post about negative experiences and feelings in order to gain social support. These associations align with previous studies suggesting that adolescents with mental health difficulties [32] and lower perceived offline social support [27, 67] use social media more frequently to secure positive feedback, emotional support, and acceptance from members of the online community. In contrast to the findings of Petrofes et al. [19], our results suggested that social media sadfishing could be considered as a response to lack of perceived social support in real life. These findings concur with the *Compensation Hypothesis* [28], which posits that online communication provides opportunities to compensate for low offline social support. However, more research is needed to examine this association.

The importance of social support in sadfishing is underscored by the finding that most adolescents in our sample never received any critical feedback (75%) or felt unsupported (65%) following sadfishing. However, positive reactions to sadfishing may be a culture-specific finding. Research shows a high prevalence of depression in Iranian teenagers [68]. The serious socio-cultural and economic challenges of the last few decades in Iran may have resulted in negative emotions being considered as normal or even ideal affective states [69] among Iranian adolescents. This may have led to increase in supportive reactions to sadfishing posts. Further research should investigate how cultural context affects reactions to sadfishing.

Despite significant correlations of social media use integration and perceived social support from significant others with social media sadfishing, these two associations did not remain significant in the regression analysis. Therefore, our second hypothesis was not confirmed. This result suggests that excessive involvement in social media is not a prerequisite for sadfishing. In other words, it seems that sadfishing is not associated with uncontrolled engagement with social media. This finding emphasizes the importance of further examining sadfishing, a phenomenon that can even be seen in occasional users of social media.

The lack of a negative association between perceived social support from significant others and sadfishing may be due to the characteristics of the dating culture in Iran [70]. Having a significant other (a romantic or sexual relationship) is uncommon among Iranian adolescents. Compared to other sources (family and friends), social support from a significant other is less available for adolescents, which results in less importance of such social support and diminished contribution to adolescent behaviors.

Additional exploratory analyses examined the associations between demographic variables and sadfishing. Results revealed higher sadfishing among boys at age 12 compared to girls. However, with advancing age, boys’ sadfishing decreased to a greater degree compared to girls. This age-gender variation in sadfishing may be related to gender stereotypes and socio-cultural restrictions in the Iranian society [71]. As boys and girls approach adolescence and face greater pressure to follow socio-cultural norms, boys may become more restrained in expressing their negative emotions in social interactions (in both offline and online social networks). Girls, in contrast, may rely more on social media for negative self-disclosure as an alternative to face-to-face disclosure. Another possible explanation may be that, with increasing age, girls face population-specific socio-cultural pressures in Iran (e.g., compulsory veiling laws and taboos around menstruation; 82). Therefore, girls may engage in social media sadfishing to disclose experiences that are not easily discussed in face-to-face interactions. Girls may be motivated to increase collective awareness of their experiences and receive social support for negative experiences. Finally, increasing sadfishing with age in girls could be attributed to a higher tendency to seek social support and a higher priority of social relationships for adolescent girls than boys [72]. However, due to the unequal gender distribution in this study, findings should be interpreted with caution regarding age-gender disparities in sadfishing. Future research should investigate how cultural differences relate to gender differences in social media sadfishing among adolescents.

This study has a number of limitations. General conclusions regarding adolescents’ sadfishing must be made with caution due to the descriptive nature of the study and convenience sampling method. Future studies should investigate sadfishing using a representative sample of adolescents. This study was conducted in Rasht, Iran. The specific social and cultural attributes of this city may not allow for making general conclusions about all Iranian adolescents based on the current findings. The socioeconomic status of participants and the most commonly used and preferred social media platforms were not recorded in the current investigation. Future studies should take these factors into account, for instance, whether adolescents with low socioeconomic status show more sadfishing behaviors and whether forms of sadfishing vary across different social media platforms. Furthermore, no information regarding parents’ attributes were collected in this investigation. The link of parents’ characteristics with adolescents’ sadfishing behavior could be investigated in future studies.The number of girls and boys participating in this study was unequal due to the use of convenience sampling. Future studies on sadfishing should use more balanced samples in terms of gender distribution.The direction of associations cannot be confirmed due to the cross-sectional study design. Moreover, the present investigation should be extended to adults in order to gain a more nuanced picture of the association of age with sadfishing. This study was conducted on an Iranian sample; therefore, cultural differences should be investigated in future studies. Previous studies have suggested that there may be considerable differences in self-disclosure on social media across cultures [73]. Finally, the *Social Media Sadfishing Questionnaire* is a self-report questionnaire, which has some disadvantages. For instance, responses may be biased due to social desirability or memory bias.

## Conclusions

Despite the limitations, the present study provides contributions to the literature on sadfishing. The construction of a brief, reliable instrument allows for the extension of previous research on sadfishing. The* Social Media Sadfishing Questionnaire* allows for the quantitative examination of sadfishing and its relationship with relevant psychological characteristics. We found that symptoms of depression, anxiety, attention seeking, and low social support predicted sadfishing among adolescents. Most adolescents reported that they never received critical feedback following their negative posts. The preponderance of supportive reactions may increase the beneficial effects of sadfishing. However, sadfishing also has the potential to increase feelings of being victimized among adolescents, and elevate the risk of negative outcomes including cyberbullying victimization [8], stalking and cyberstalking, sexual abuse, criminal exploitation, government surveillance, and interpersonal harms [74]. Therefore, future studies should explore both the positive and negative consequences associated with sadfishing.

## Data Availability

The data are available from the corresponding author on reasonable request.
